# Palliative and End-of-Life Care Conversations with Older People with Chronic Obstructive Pulmonary Disease in Croatia—A Pilot Study

**DOI:** 10.3390/healthcare8030282

**Published:** 2020-08-20

**Authors:** Petra Čičak, Sanja Thompson, Sanja Popović-Grle, Vladimir Fijačko, Jasmina Lukinac, Ana Marija Lukinac

**Affiliations:** 1 Faculty of Medicine, Josip Juraj Strossmayer University of Osijek, 31000 Osijek, Croatia; fijacko.vladimir@gmail.com (V.F.); lukinac28@gmail.com (A.M.L.); 2 Department of Pulmonology, University Hospital Center Osijek, 31000 Osijek, Croatia; 3 Department of Clinical Geratology, John Radcliffe Hospital, University of Oxford, Oxford OX3 9DU, UK; Sanja.Thompson@ouh.nhs.uk; 4 Clinical Department for Lung Diseases Jordanovac, University Hospital Center Zagreb, School of Medicine University of Zagreb, 10000 Zagreb, Croatia; spopovi1@kbc-zagreb.hr; 5 Faculty of Food Technology Osijek, Josip Juraj Strossmayer University of Osijek, 31000 Osijek, Croatia; ptfosptfos2@gmail.com; 6 Department of Rheumatology, Clinical immunology, Allergology, University Hospital Center Osijek, 31000 Osijek, Croatia

**Keywords:** COPD, end of life care, palliative care, communication

## Abstract

Despite the progressive nature of chronic obstructive pulmonary disease (COPD), its association of high morbidity and mortality with severe COPD, and the view that discussions between patients and clinicians about palliative care plans should be grounded in patients’ preferences, many older patients do not receive timely end-of-life care (EOLC) discussions with healthcare professionals (HPs), potentially risking inadequate care at the advanced stages of the disease. The aim of this pilot study was to evaluate EOLC discussions and resuscitation issues as a representative and illustrative part within EOLC in older patients with COPD in the University Hospital Center Osijek, Slavonia (Eastern Region), Croatia, as such data have not yet been explored. The study was designed as cross-sectional research. Two groups of participants, namely, patients at least 65 years old with COPD and healthcare professionals, were interviewed anonymously. In total, 83 participants (22 HPs and 61 patients with COPD) were included in the study. According to the results, 77% of patients reported that they had not had EOLC discussions with HPs, 64% expressed the opinion that they would like such conversations, and the best timing for such discussion would be during frequent hospital admissions. Furthermore, 77% of HPs thought that EOLC communication is important, but only 14% actually discussed such issues with their patients because most of them felt uncomfortable starting such a topic. The majority of older patients with COPD did not discuss advanced care planning with their HPs, even though the majority of them would like to have such a discussion. EOLC between HPs and older patients with COPD should be encouraged in line with patients’ wishes, with the aim to improve their quality of care by anticipating patients’ likely future needs in a timely manner and thereby providing proactive support in accordance with patients’ preferences.

## 1. Introduction

Chronic obstructive pulmonary disease (COPD) is currently the only chronic disease to show a significant increase in mortality. By 2040, COPD is projected to become the fourth most common cause of mortality worldwide [1,2]. It is a common, slowly progressive respiratory disease, characterized by persistent respiratory symptoms and limited airflow. In advanced stages, the disease is manifested by an increase in exacerbations and hospital admissions, resulting in a poor quality of life for these patients [3]. Nevertheless, many older patients do not receive adequate palliative care and do not have a timely end-of-life care (EOLC) discussions with healthcare professionals (HPs) [4]. Palliative care is a broader term that includes EOLC, which is focused on improving quality of life and minimizing symptoms before the end-of-life period [5]. It is suggested that effective palliative care should involve open communication between HPs and patients. EOLC issues, such as patients’ hopes and fears, place of death, and prognosis, need to be discussed [6]. The palliative and EOLC needs of older patients with COPD are rarely discussed, mainly due to HPs’ view that these discussions, though necessary, are difficult. They are more likely to have such discussions with cancer patients than those with severe COPD, believing that only a minority of patients would want to know their prognosis [7,8]. Most patients with COPD prefer treatment focused on comfort rather than on prolonging life, and patients with COPD are equally as likely as lung cancer patients to prefer not to be intubated or receive cardiopulmonary resuscitation [9]. Furthermore, the majority of HPs feel uncomfortable approaching the EOLC discussion and are unsure when to initiate it [10,11]. The uncertainty and difficulties in predicting the prognosis for patients make communication about EOLC more difficult [8,10]. Patients with advanced COPD experience a prolonged deterioration of lung function with low quality of life, uncontrolled symptoms, psychological morbidity, social isolation, and unmet communication and information needs. The quality of life of patients with COPD appears to be at least as poor or even worse than patients with lung cancer [7,11,12]. Patients with COPD have a 34% higher risk of sudden cardiac death when compared with people of the same age and sex without the disease. Their risk almost doubles more than five years after first being diagnosed with COPD [13]. EOLC refers to the healthcare of patients with a terminal illness or terminal condition and implies the humane and respectful care of patients and their close family members. Adequate communication between patients and HPs is a key aspect affecting the quality of care of dying patients. Therefore, poor management of health information and communication at the end of life increase the suffering and discomfort of these patients [14,15]. HPs should take the initiative and discuss patient goals for EOLC or palliative care. HPs can promote communication, education, and discussion related to EOLC and its implications on the patient and their families in order to facilitate improved decision-making. Effective advanced planning can assist with putting forth the patient’s autonomous choices [16].

This pilot study investigated older patients with COPD and their clinicians’ present practice in EOLC communication/palliative care in Croatia, as such data are unknown at present, as well as the cause of poor communication about EOLC. The topic of resuscitation was chosen for our questionnaire as a representative and illustrative part within EOLC that allowed the patient to realize the complex issue as vividly as possible.

## 2. Materials and Methods 

**Figure 1:**
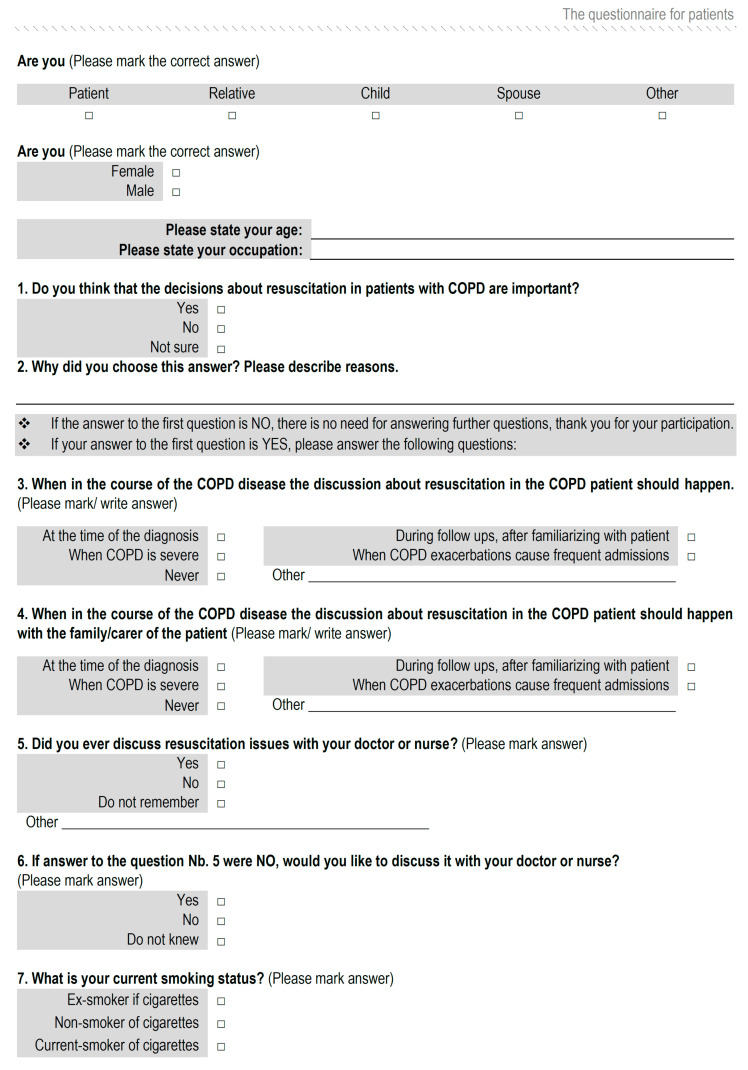
The questionnaire for patients. COPD: Chronic obstructive pulmonary disease.

This study was conducted in the Department of Pulmonology, University Hospital Center Osijek, Croatia, between February 2018 and October 2018. The study was designed as a cross-sectional study. There were two groups of participants, namely, patients with established diagnoses of COPD and their HPs. The Global Initiative for Obstructive Lung Disease (GOLD) system categorizes airflow limitation severity in COPD into stages (based on the post-bronchodilator forced expiratory volume over 1 s), with the mildest form being stage 1 and the very severe form being stage 4 [17]. Patients with COPD who were included in the study were diagnosed with stage 3. Ethical approval was granted by the Ethical Committee of University Hospital Center Osijek (at its session no. R2-4718/2018, held on 29 May 2018). Participants were asked by the investigator (first author) about their willingness to participate in the research and were handed an information leaflet and a self-administered questionnaire. The patients eligible for the study had a diagnosis of COPD, were at least 65 years old, and gave verbal consent for participation in the study. The exclusion criteria for patients with COPD were: (1) younger than 65 years; (2) the inability to read, write, and communicate; (3) a score of less than 6 in the abbreviated mental test score (AMTS); (4) patients with a GOLD stage other than stage 3 at the time of the study initiation; and (5) patients who did not provide verbal consent to participate. Patients were asked to complete a self-administrated questionnaire (Figure 1) that contained various questions relating to their previous experience about EOLC communication with HPs related to COPD and future wishes related to such conversations. The topic of resuscitation was chosen in this questionnaire as a representative and illustrative part within EOLC that allowed the patient to realize the complex issue as vividly as possible. Before joining the research, the AMTS was carried out on all patients [18]. Those who had a score less than 6 were not included in the study, and the remaining patients and HPs received an information leaflet in which the goal, purpose, and benefit of this research were explained. The nature and type of the questions and the topic to be discussed in the questionnaire were explained. It was stated that there was no risk of a breach of confidentiality. Participation was voluntary, anonymous, and the questionnaire was completed only once. The ethical committee approved verbal consent as an alternative to the written form because this research involved a minimal risk for all participants.

**Figure 2:**
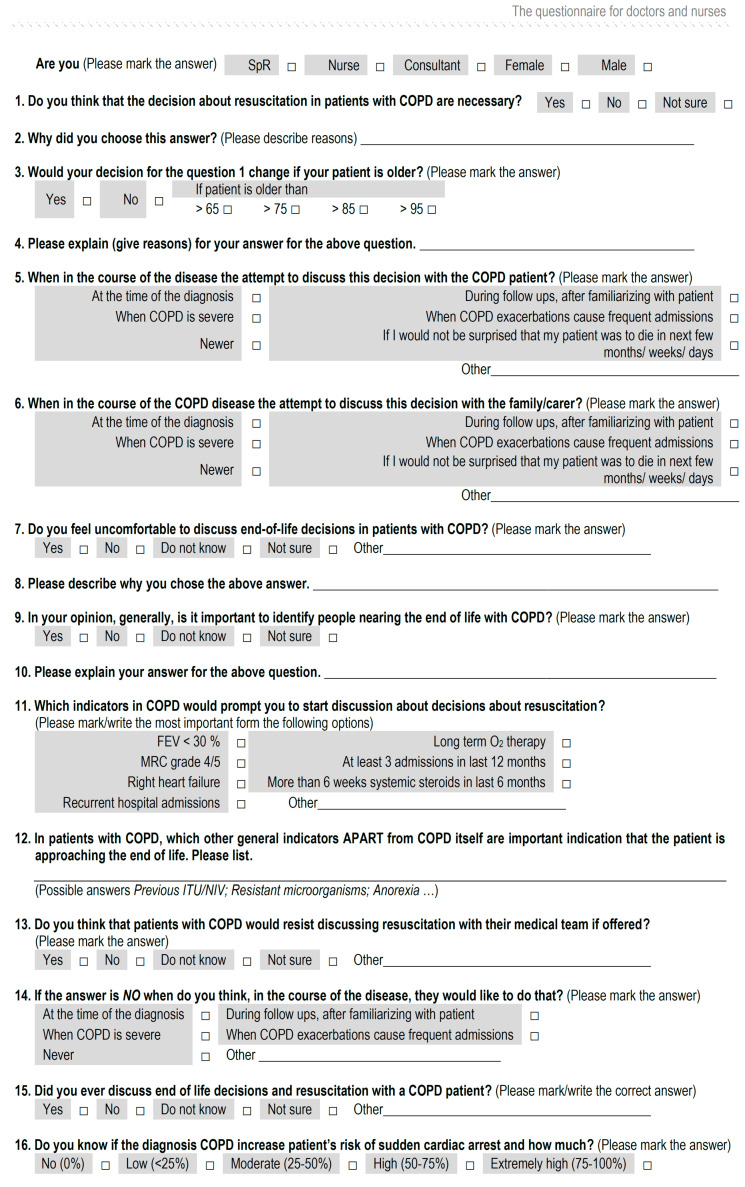
The questionnaire for doctors and nurses. FEV: Forced expiratory volume, ITU: Intubation, MRC: Medical Research Council, NIV: Non-Invasive Ventilation, SpR: Specialist registrar.

HPs included in the study were specialist registrars, consultants (senior hospital-based physicians), and nurses based at the pulmonology department at the University Hospital, Osijek. Purposive sampling strategies were used to maximize the variation in the sample and experience related to EOL care. We focused on all HPs in our clinic who were involved in the everyday care of patients with COPD to explore their opinions and get a holistic view of this issue. The exclusion criteria for HPs were: (1) less than six months’ experience in the pulmonology department and (2) did not provide verbal consent to participate. They were asked about their attitudes and opinions on EOLC communication/palliative care in older patients with COPD. There was a mix of multiple-choice questions and open-ended questions (Figure 2). Patients were identified through outpatient pulmonary clinics, as well as during their in hospital admission. Every eligible patient was approached to participate in the study. A total of seven subjects refused to participate, six patients and one HP. Due to the AMTS, an additional four patients were excluded. The participation rate was 88%. Survey data were described via descriptive statistics using the SPSS Statistics V25.0 software package (IBM Corp., Armonk, N.Y., USA). Descriptive data were expressed in frequency and content for a nominal variable. Numerical variables with a normal distribution were described with a mean and standard deviation. 

## 3. Results

Two groups of participants, namely, patients at least 65 years old with COPD (*N* = 61) and HPs (*N* = 22), were included in the study. The mean age of patients with COPD was 75 years; 42 male (mean age 75, youngest 66, oldest 85) and 19 female (mean age 75, youngest 67, oldest 86) patients. The results showed that the majority of patients had primary and secondary education. The majority of patients were ex-smokers (*N* = 45), while the rest were current smokers (Table 1).

The comorbidities were reported in 84% (*N* = 51) of all patients. The most common comorbidities in patients were arterial hypertension 79% (*N* = 48), atrial fibrillation 26% (*N* = 16), and diabetes mellitus 25% (*N* = 15) (Table 2).

**Figure 3:**
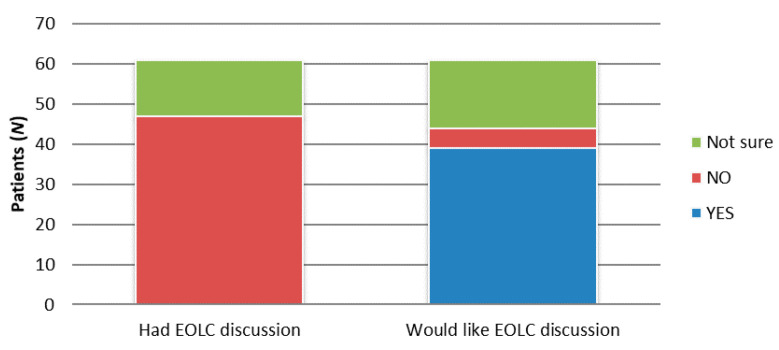
End-of-life care (EOLC) discussion that took place and patients’ preferences about such discussion.

**Figure 4:**
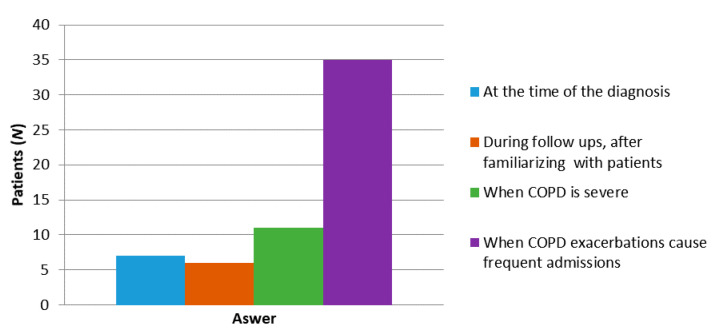
Patients’ preferences regarding the timing of the EOLC discussion.

The majority, 77% (*N* = 47), reported that they had not had EOLC discussions with HPs, and the majority, 64% (*N* = 39), would like such a conversation. Only 8% (*N* = 5) of patients were not keen on such a discussion (Figure 3). A total of 67% (*N* = 42) of the interviewed patients thought that EOLC is important and that such a conversation should take place, and according to the majority, 53% (*N* = 32) think that the best time for this is during frequent hospital admissions (Figure 4).

Among the 22 HPs included in this study, 32% (*N* = 7) were consultants in pulmonology, 36% (*N* = 8) were specialist registrars in pulmonology, and 32% (*N* = 7) were nurses from the pulmonology department (Table 1). The majority of them did not discuss advanced care planning with their patients with COPD, including any kind of patient–clinician communication about EOLC. The majority of them, 77% (*N* = 17), thought that this topic was important, but only a minority 14% (*N* = 3) discussed these issues with their patients. The main reason was feeling uncomfortable to start such a topic. In our study, we investigated the HPs’ awareness regarding sudden cardiac death in patients with COPD. When asked, all of them answered positively, and most of them identified a moderate risk of sudden cardiac death in patients with COPD. The question was directed to the HPs to examine their knowledge and awareness of the difficulty in estimating patients’ life expectancy with COPD. The aim of this question was to examine the need for additional education of HPs to anticipate and initiate timely EOLC communication. All HPs were unanimous when asked about the importance of identifying people nearing the end of life with COPD and answered positively. On the other hand, the respondents were not unanimous when asked about the indicators in COPD that would prompt the HPs to start a discussion about resuscitation. Right heart failure (RHF) and recurrent hospital admissions were identified as almost equally important, while forced expiratory volume over 1 s (FEV1) < 30% and long-term oxygen therapy (LTOT) were less recognized as indicators.

The HPs acknowledged the value of EOLC conversations, but when asked whether their patients would decline such a conversation, the majority were unsure (64%), while only 36% (*N* = 8) gave a negative answer. In addition, when asked about the timing of a conversation about EOLC, most HPs anticipated an advanced stage of the disease to be the “patient’s ideal timing” for such a conversation (Table 3).

## 4. Discussion

COPD is estimated to be the fourth highest cause of death worldwide by 2040 [2]. For many patients, maximal therapy for COPD produces only a modest relief of symptoms, leaving patients with significantly reduced health-related quality of life [1]. Despite the disease’s progressive nature, many older patients receive inadequate palliative care, as current care practices are not facilitating satisfactory and timely discussion about palliative care between patients and their HPs [4,19]. The aim of this pilot study was to evaluate the information communicated about EOL and palliative care in older patients with COPD in a regional University hospital in Croatia (Slavonia), which cares for the population of approximately 805,000 people in the Eastern region, as such data have not been explored yet. Of the interviewed patients with COPD, 77% did not have EOLC discussions with HPs, and 64% of the interviewed patients would like such a conversation. Only a minority, 8% of patients, were not keen on such a discussion. A study by Curtis et al. [4] showed that only a third of patients with COPD with severe disease had discussed EOLC with their HPs. Four previous reviews have also shown that only a minority of patients with severe COPD have discussed EOLC issues with their HPs [20,21,22]. The reason for this is due to the difficulty in predicting illness progression, making it hard for HPs to define the right timing for such a conversation. Many HPs find conversations initiated by patients easier, still admitting to feeling uncomfortable when a patient asks about EOLC directly [8,10,23]. In our study, the main reason was that HPs were uncomfortable starting such a topic.

Regarding prognostication challenges, HPs should be encouraged to identify patients with COPD for whom EOLC discussions are especially important. A special profile emerges of patients at high risk of mortality and morbidity by taking into account disease indicators identified in previous studies: FEV1 < 30%; LTOT; one or more hospital admissions in the previous year for an acute exacerbation of COPD; left heart failure or other comorbidities, such as weight loss or cachexia; age > 70 years; and decreased functional status and increasing dependence on others [5,24]. In our study, HPs were asked which of those abovementioned indicators would prompt them to start EOLC discussions with their patients. Most of them thought that right heart failure and recurrent hospital admissions were the most important indicators. When asked about EOLC importance, most HPs thought this topic is important, but only a minority had had such a conversation with their patients. A study by Elkington et al. showed that HPs are more likely to have such discussions with cancer patients than those patients with COPD, believing that only a minority of patients want to know their prognosis and it is difficult to recognize who these individuals are [7].

Our study identified that two-thirds of interviewed patients believed that the EOLC is important, and most of them thought that the best time for this conversation is during frequent hospital admissions, similar to the findings in other studies [25,26]. In our study, most HPs anticipated an advanced stage of the disease in the form of right heart failure, recurrent hospital admissions, and LTOT as the “patient’s ideal timing” for such a conversation. Interestingly, neither the patients nor the HPs suggested that such discussions should take place at an early stage of the disease [27].

## 5. Conclusions

Our current practices do not facilitate satisfactory conversations about palliative and EOL care in older patients with severe COPD. The majority of older patients with COPD did not have the chance to discuss advanced care planning with their HPs, including any kind of patient–clinician communication about EOLC. Improving communication represents an important opportunity for the improvement of the quality of COPD care in these patients. The majority of HPs interviewed felt uncomfortable approaching such a discussion, while the majority of older patients would like to have such a discussion. EOLC discussions with the older patients with advanced COPD should be encouraged between HPs and their patients in line with the patients’ wishes, aiming to improve their quality of care, as anticipating patients’ likely future needs in a timely manner makes it possible to provide proactive support in accordance with patients’ preferences. We strongly believe that this study is another keystone in providing proof that EOLC communication and palliative care are a global issue that is not linked to specific geographic regions or cultures, adding value in the global perception.

### Limitations of the Study

The main limitation was the number of participants, especially HPs. The reason for this lay in the fact that the study was conducted at one clinic in one region of our country. 

## Figures and Tables

**Table 1:** Survey participants’ data.

Characteristics	*N*	%	Mean (SD)	Min	Max
Patients					
Age					
Female	19	31.1	75.2 (5.1)	67	86
Male	42	68.9	74.8 (4.8)	66	85
Total	61	100.0	75.0 (5.1)	66	86
Education					
Elementary school	28	45.9			
High school	30	49.2			
Higher education	3	4.9			
Smoking Status					
Current smoker	16	26.2			
Ex-smoker	45	73.8			
Total	61	100.0			
Healthcare Providers					
Professional Level					
Nurse	7	31.8			
Specialist registrar	8	36.4			
Consultant	7	31.8			

**Table 2:** Comorbidity data.

Disease	*N*	%
Arterial hypertension	48	78.7
Atrial fibrillation	16	26.2
Diabetes mellitus	15	24.6
Renal insufficiency	4	6.6
Hypothyroidism	2	3.3

**Table 3:** EOLC data regarding HPs (*N* = 22).

Characteristics	Value	*N*	%
HPs reports regarding the importance of EOLC discussion	Not important	5	22.7
	Important	17	77.3
Do you discuss these issues with your patients with COPD?	Yes	3	13.6
	No	19	86.4
HPs reports regarding the ideal timing to start EOLC discussions with patients with COPD	At the time of the diagnosis	2	9.1
	During follow-ups, after familiarizing with the patient	2	9.1
	When COPD is severe	9	40.9
	When COPD exacerbations cause frequent admissions	8	36.4
	Never	1	4.5
In your opinion, is it generally important to identify people nearing the end of life with COPD?	Yes	22	100.0
Which indicators in COPD would prompt you to start a discussion about resuscitation?	FEV1 * < 30%	2	9.1
	LTOT **	3	13.6
	RHF ***	9	40.9
	Recurrent hospital admissions	8	36.4
Do you know whether the diagnosis of COPD increases a patient’s risk of sudden cardiac arrest and by how much?	No risk (0%)	0	0
	Low risk (<25%)	2	9.1
	Moderate risk (25–50%)	12	54.5
	High risk (50–75%)	7	31.8
	Extremely high risk (75–100%)	1	4.5

*—Forced expiratory volume over 1 s, **—Long-term oxygen therapy, ***—Right heart failure.
